# Recent Progress of Chiral Mesoporous Silica Nanostructures: From Synthesis to Applications

**DOI:** 10.3390/molecules30224455

**Published:** 2025-11-19

**Authors:** Changlong Hao

**Affiliations:** State Key Laboratory of Food Science and Resources, School of Food Science and Technology, Jiangnan University, Wuxi 214122, China; hcl@jiangnan.edu.cn

**Keywords:** chirality, mesoporous silica nanostructures, synthesis, applications

## Abstract

Chiral mesoporous silica nanostructures (MSNs) have emerged as a cutting-edge material in nanotechnology. These nanostructures not only retain the tunable physicochemical properties of traditional MSNs—such as adjustable pore size, high surface area, and excellent biocompatibility—but also exhibit unique functionalities and biological behaviors due to their helical architectures at both molecular and macroscopic levels. This inherent chirality grants chiral MSNs exceptional potential in diverse applications, including chiral catalysis, enantiomeric separation, chiral recognition, and advanced drug delivery systems. Over the past five years, substantial progress has been made in understanding their synthesis mechanisms and practical applications. This review provides a comprehensive analysis of recent advancements in chiral silica nanostructures, with a focus on the synthesis strategies and applications of chiral MSNs. Emphasis is placed on their roles in chiral recognition, drug delivery, chiral separation, nanomedicine, and asymmetric catalysis. By highlighting these developments, this review serves as a roadmap for the rational design and translational applications of chiral silica nanostructures, offering valuable guidance for unlocking their full potential.

## 1. Introduction

Chirality, a fundamental characteristic describing structures that cannot be aligned with their mirror reflections, was initially identified by Louis Pasteur in his research on tartaric acid isomers. This form of molecular asymmetry is widely observed in biological systems, manifesting in key compounds such as amino acids, sugars, proteins, and DNA. For instance, among the 20 proteinogenic amino acids, all except glycine have an L-form (levorotatory) configuration, while sugars and deoxyribose in biological systems uniformly adopt the D-form (dextrorotatory) configuration. Additionally, biological structures such as plant tendrils and mollusk shells display inherent chirality. Chirality plays a crucial role in biological systems, as chiral compounds often exhibit significant differences in pharmacological effects, metabolic pathways, toxicity, and therapeutic efficacy due to enantiomeric variations. For example, stereoisomers in pharmaceuticals can lead to distinct biological responses, metabolic profiles, and therapeutic outcomes.

Chiral nanostructures have garnered significant attention due to their favorable interactions with chiral molecules [1,2,3,4,5]. Mesoporous silica, known for its tunable physicochemical properties (e.g., pore size, surface area), biocompatibility, and biodegradability, has emerged as an important material in this field. Chiral mesoporous silica nanostructures (MSNs), which are either modified with chiral agents or feature helical channels, represent a prominent subclass of chiral nanomaterials. These materials combine high surface area, superior connectivity, strong adhesiveness, and high bioresponsiveness with chiral topologies at both macroscopic and molecular scales [6,7,8]. Such properties make them highly valuable in applications such as drug delivery, asymmetric catalysis, and chiral separation.

While the field of chiral nanomaterials has been extensively reviewed, including notable contributions such as the broad overview of chiral inorganic nanostructures by Govan et al. [1] and the specialized focus on template design for chiral MSNs by Yin et al. [2], this review is dedicated to providing a timely and comprehensive update exclusively on chiral mesoporous silica nanostructures over the past five years. Distinct from previous works, our emphasis is placed on the interplay between novel synthesis strategies and their direct implications in cutting-edge applications. We particularly highlight areas that have witnessed significant recent growth, such as the enantioselective oral absorption of drugs, the therapeutic potential in treating neurodegenerative (e.g., Alzheimer’s disease) and inflammatory diseases (e.g., IBD), and the innovative use of chiral MSNs as templates for engineering circularly polarized luminescence in perovskite nanocrystals. By consolidating these most recent advances and critically discussing the structure-property-application relationships, this review aims to serve as an up-to-date and focused resource, offering valuable insights for researchers navigating the rapidly evolving landscape of chiral silica nanomaterials.

In this review, we systematically examine recent breakthroughs in the synthesis and applications of chiral silica nanostructures, with a focus on progress made in the last five years. We begin by providing a concise overview of advances in synthetic methodologies. This is followed by a detailed discussion of emerging applications in drug delivery, disease therapy, chiral recognition, separation, and asymmetric catalysis, which remain in their early stages of development. Lastly, we offer insights into potential future applications and address key concerns, such as toxicity and biocompatibility. By doing so, this review provides valuable guidance for the design and application of chiral silica nanostructures, offering significant reference value for researchers and industry professionals alike.

## 2. Synthesis Strategy

The synthesis of chiral silica nanostructures (such as chiral mesoporous silica films [9] chiral mesostructured silica nanofibers [10])—an intricate process combining chiral chemistry and nanomaterial science—focuses on introducing chirality into silicon nanomaterials [9,10,11,12,13,14]. Current mainstream methods are broadly divided into two categories: the template-directed method and the chiral ligand-induced method. Each employs distinct mechanisms to achieve precise control over chiral structures. These strategies have significantly advanced structural modulation and performance enhancement in chiral silica nanostructures.

### 2.1. Template-Directed Method

**Figure 1:**
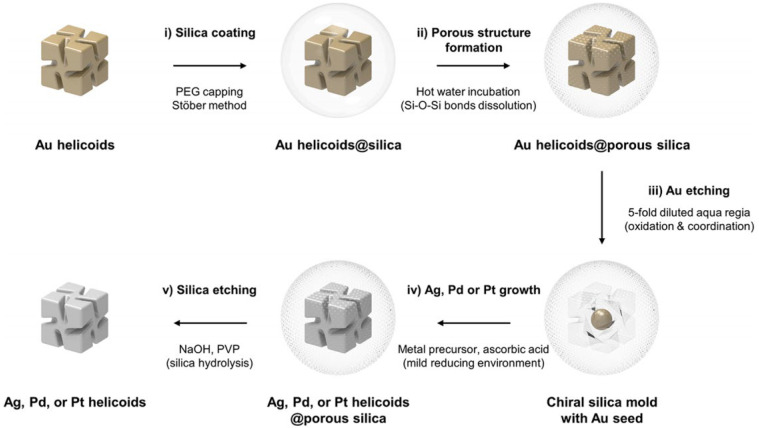
Graphical representation of the overall synthetic process of chiral silica mold and chiral Ag, Pd, or Pt helicoids. Reproduced from ref. [18] with permission from the ACS.

The template-directed method, the most widely used approach, involves two primary techniques: hard-template methods and soft-template methods [15,16,17]. The hard-template method utilizes a hard template with a specific chiral structure as a guiding framework. Silica nanostructures are formed by infiltrating a silicon precursor into the template, followed by template removal. The process typically involves three steps: template preparation, precursor deposition, and template removal. Incorporating a chiral-directing mechanism during template design is crucial for constructing chiral structures. In 2024, Nam’s group used pre-synthesized rigid templates, such as chiral gold helices, to guide the growth of silicon precursors. After etching the gold helices, they obtained chiral silica molds (Figure 1) [18]. These molds acted as geometric constraint reactors, enabling the synthesis of chiral metallic helices, including Ag, Pd, and Pt. The generation of chirality in hard-templating is fundamentally a geometric transcription process. The pre-synthesized chiral metal helix (e.g., Au) provides a rigid, nanoscale chiral interface. During the infiltration of the silica precursor, this interface acts as a steric constraint, directing the condensation of silica into a complementary, inverse chiral mold. The chirality is preserved upon template removal because the silica network forms a mechanically robust, negative replica. The fidelity of this transcription depends critically on the conformational stability of the template and the conformity of the silica precursor during deposition and curing. Unlike soft templates, this method does not rely on molecular-level chiral interactions but on the top-down replication of a pre-defined chiral morphology.

**Figure 2:**
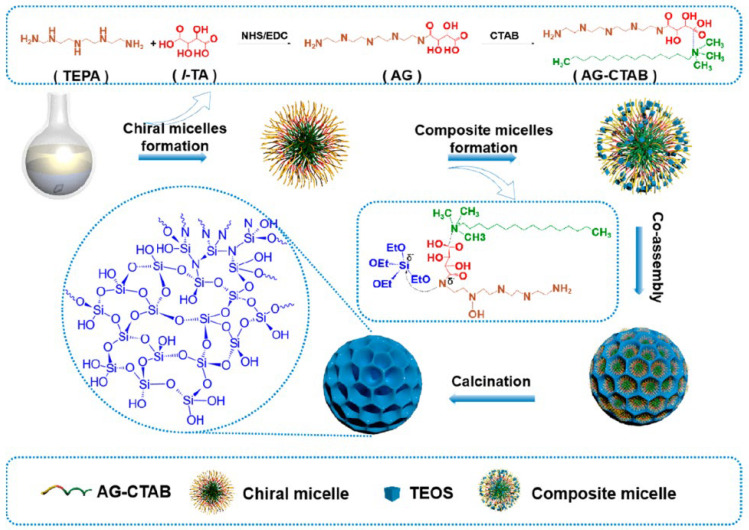
Schematic illustration for the self-assembly synthesis of the L-mSiO_2_ nanospheres by using the designed micelles with chiral and NH_2_−groups as the building blocks for organic–inorganic self-assembly. Reproduced from ref. [19] with permission from the ACS.

This soft-template method relies on chiral surfactants or supramolecules to create helical pores or chiral mesoporous structures through self-assembly. Chiral surfactants interact with silica precursors via electrostatic, hydrogen-bonding, or hydrophobic interactions, resulting in porous structures with defined chirality. Zhao’s group developed a chiral amide-gel-directed synthesis strategy to prepare mesoporous silica nanoparticles with molecular-scale chiral silicate skeletons (Figure 2) [19]. The process involved: (I) Forming nearly pure chiral amide gels (AG) by cross-linking amine-containing tetraethylenepentamine (TEPA) with carboxyl-containing tartaric acid (TA), (II) creating micelles from AG and cetyltrimethylammonium bromide (CTAB) via hydrogen bonding, (III) hydrolyzing and condensing tetraethyl orthosilicate (TEOS) around these micelles, forming ordered mesoporous structures. The charged interactions between amine and carboxyl groups in the micelles played a critical role in alkoxysilane hydrolytic polycondensation. Finally, the organic templates were removed by calcination at 700 °C, yielding the final chiral MSNs.

In soft-templating approaches, chirality emerges from the supramolecular self-assembly of chiral directing agents. For instance, in the chiral amide-gel system [19], the cross-linking between TEPA and TA creates a chiral supramolecular scaffold. The subsequent incorporation of CTAB and TEOS is guided by a combination of hydrogen bonding and electrostatic interactions. The chirality of the molecular building blocks (TA) is amplified to the mesoscale through these cooperative non-covalent forces, leading to the formation of a helical micellar architecture. The silica precursor then condenses around this chiral co-assembly, ‘freezing’ the transient supramolecular chirality into a permanent, rigid silica skeleton. The key mechanistic insight is that the final mesoscopic chirality is a result of the chiral molecular information being transmitted and amplified through the self-assembly pathway.

Biopolymers such as DNA [20], and peptides [21,22], which naturally form chiral helical conformations through non-covalent interactions [23], offer promising pathways for synthesizing chiral silica materials [24,25,26,27]. Chiral mesostructured silica can be prepared by self-assembling DNA, metal ions, and silica sources. For example, Manabe et al. demonstrated a method to produce silica materials with preferred-handed helical structures [28]. The process involved: (I) Synthesizing isotactic poly(methacrylate)-functionalized polyhedral oligomeric silsesquioxane (it-PMAPOSS) with stereo-regularity via living anionic polymerization, (II) mixing it-PMAPOSS with chiral dopants, such as (S)-(−) or (R)-(+)-binaphthol, to induce helical conformations, (III) removing the dopants through heat treatment, while preserving the chiral helical structure within the silica material.

A unified mechanistic framework for biomolecular templates (e.g., DNA, peptides) can be constructed based on their inherent hierarchical chirality and specific interactions. These biopolymers possess well-defined secondary structures (e.g., DNA double helix, α-helix, β-sheet) that serve as ‘chiral seeds’. During silica mineralization, electrostatic attraction between the negatively charged silica species (silicic acid) and positively charged groups on the biopolymer (e.g., protonated amines in PEI or peptide side chains) initiates the interaction. This is complemented by hydrogen bonding between silanol groups and the biopolymer’s backbone, which aligns the silica precursor along the chiral axis of the template. Metal ions (e.g., Ca^2+^, Mg^2+^) often act as ionic bridges, facilitating the condensation process. Thus, the mesoscale chiral architecture of the final silica material is a direct biomimetic transcription of the template’s nano-scale and molecular-scale chirality, governed by a combination of ionic, hydrogen bonding, and stereochemical complementarity.

### 2.2. Chiral Transfer Strategy

**Figure 3:**
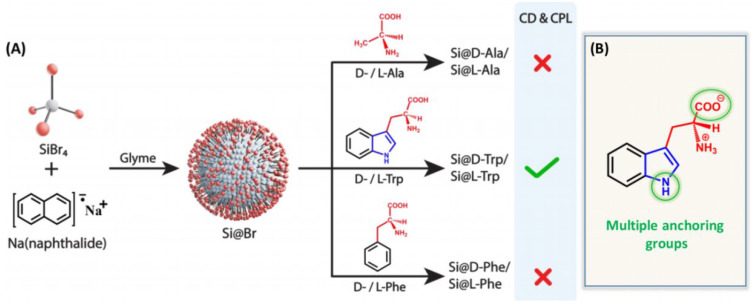
(**A**) Synthesis of silicon nanoparticles capped with various ligands: D- and L-isomers of alanine (Ala), tryptophan (Trp), and phenylalanine (Phe). (**B**) Representation of multiple anchoring groups in Trp (nitrogen-containing electron-rich indole ring and the carboxylate group), which can directly bind on the Si surface and provide chirality. Reproduced from ref. [30] with permission from the ACS.

**Figure 4:**
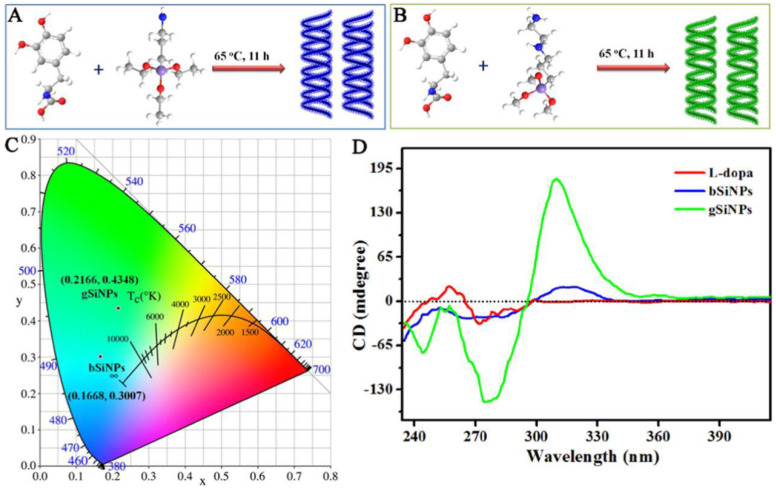
Schematic diagram of the synthesis of chiral (**A**) blue-emitting silicon nanoparticles (bSiNPs) and (**B**) green-emitting silicon nanoparticles (gSiNPs). (**C**) CIE coordinates of the chiral bSiNPs and gSiNPs. (**D**) CD spectra of L-DOPA, chiral bSiNPs, and gSiNPs. Reproduced from ref. [31] with permission from the ACS.

Another approach for synthesizing chiral silicon nanomaterials involves molecular-level chirality induction [28,29]. This method uses chiral ligands to transfer their chirality to silicon nanomaterials. For example, Sujith et al. employed a nucleophilic substitution reaction to synthesize water-soluble chiral silicon nanoparticles (SiNPs) functionalized with D- and L-tryptophan at room temperature (Figure 3) [30]. The chiral optical response was attributed to multiple interactions between tryptophan and the nanoparticle surface, involving the indole nitrogen and -COO^−^ groups. These interactions transferred the enantiomeric structural imprint to the silicon surface. However, phenylalanine- and alanine-modified SiNPs, which lacked Si–N bonds or chiral features, did not exhibit chiral optical properties. This highlights the structural requirements for ligand-induced chirality in silicon nanomaterials. Notably, this study was the first to report circularly polarized luminescence in silicon nanoparticles, paving the way for next-generation silicon-based optical systems. Using L-DOPA as a chiral source, two types of chiral SiNPs with distinct fluorescence emission wavelengths were synthesized (Figure 4) [31]. By selecting different silicon precursors, researchers produced blue-emitting SiNPs (bSiNPs) and green-emitting SiNPs (gSiNPs). Circular dichroism (CD) spectra confirmed that both bSiNPs and gSiNPs exhibited Cotton effects consistent with L-DOPA in the 240–296 nm range, indicating successful chirality transfer. The absolute quantum yields for bSiNPs and gSiNPs were 0.3% and 1.9%, respectively.

Beyond the specific examples described above, the efficacy of chirality transfer from molecular ligands to silica nanomaterials is governed by several unifying principles that bridge the molecular and mesoscopic scales. First, the strength and multidentate nature of the surface-ligand interaction are paramount; high-fidelity chirality transfer, as observed with tryptophan, often requires strong, multipoint anchoring (e.g., simultaneous Si–N bonding and electrostatic interactions) to effectively imprint the chiral conformation onto the nanoparticle surface. Second, the intrinsic rigidity of the chiral ligand itself influences its ability to act as a stereochemical template. Rigid, conformationally restricted molecules more effectively transmit and maintain their chiral information compared to flexible ligands, which are susceptible to conformational fluctuations that can dissipate the chiral signal. Finally, in systems where ligands can self-assemble, a supramolecular chirality amplification mechanism can occur. Here, the initial chiral induction from a single molecule is cooperatively enhanced through non-covalent interactions (e.g., hydrogen bonding, π-π stacking) within a supramolecular structure, leading to a collective chiroptical response that is significantly stronger than the sum of individual molecular contributions. Understanding these factors—interaction strength, ligand rigidity, and supramolecular cooperativity—provides a predictive framework for designing chiral silica nanomaterials with tailored optical properties.

### 2.3. Comparative Analysis of Synthesis Strategies

The choice of synthesis method fundamentally determines the structural characteristics and consequently the functional performance of chiral MSNs. Table 1 provides a systematic comparison of the major synthesis strategies, highlighting their distinctive features, advantages, and limitations. The selection of an appropriate synthesis strategy for chiral MSNs involves critical trade-offs between structural precision, functional performance, and practical feasibility. Template-directed methods, particularly hard templating, offer unparalleled control over chiral geometry at the nanoscale, making them ideal for applications requiring precise optical properties or specific nanomolding. However, this precision comes at the cost of complex multi-step processes and challenges in template removal and scalability. Soft templating provides a more versatile platform with greater synthetic flexibility and often better suitability for biomedical applications, though it demands careful optimization of reaction conditions. In contrast, chiral transfer via molecular grafting represents the most straightforward approach for creating water-compatible systems but typically yields weaker chiral signals due to the absence of long-range order. For applications prioritizing strong chiroptical responses, template-directed approaches are generally preferred, while molecular grafting may suffice for biological sensing where water solubility and simplicity are paramount. Ultimately, the choice of synthesis method should be guided by the specific application requirements, balancing factors such as structural complexity, optical activity, biological compatibility, and manufacturing scalability.

## 3. Application of Chiral Silica Nanostructures

Chiral silica nanostructures have gained attention for their remarkable properties, offering diverse applications in chiral recognition, separation, and environmental remediation. These applications leverage the unique chiral topological structures, tunable pore characteristics, and surface modification capabilities of chiral silica nanostructures.

### 3.1. Applications in Chiral Recognition

**Figure 5:**
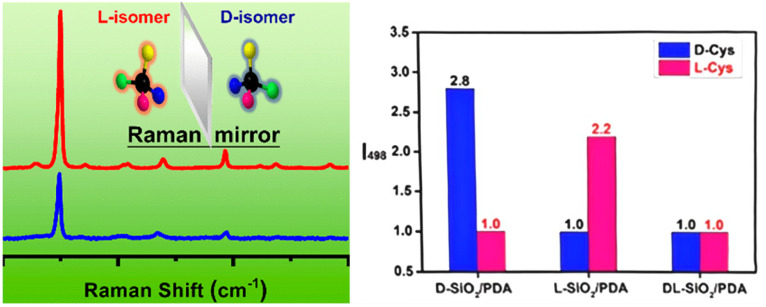
Schematic illustration the detection of enantiomers using chiral SiO_2_/PDA, and the relative values of I_498_ (Raman intensity at 498 cm^−1^) for Cys enantiomers mixed with D-, L-, and DL-SiO_2_/PDA. Reproduced from ref. [34] with permission from the ACS.

Chiral MSNs and chiral silica gels enable highly selective molecular recognition through their chirality-induced structural features and functionalized surfaces [31,32,33,34]. While chiral MSNs and silica gels leverage their stereochemical properties for enantioselective recognition, the analytical characterization of such interactions often relies on spectroscopic techniques. However, a critical challenge arises with conventional methods like Raman spectroscopy, which, despite offering molecular vibrational fingerprints with high sensitivity and non-destructive analysis, fails to distinguish enantiomers due to their identical spectral signatures. Kong et al. addressed this limitation by designing chiral-selective functional materials [34]. They exploited the ability of chiral silica to induce differential Raman scattering signals between enantiomers, achieving enantioselective recognition. Their process involved fabricating chiral SiO_2_ nanofibers through a template-directed sol–gel method. Firstly, catalytic chiral templates, composed of polyethyleneimine (PEI) and either D- or L-tartaric acid (TA), were prepared. Then these templates directed the hydrolysis and condensation of tetramethoxysilane, forming PEI/TA@SiO_2_ hybrid materials. Subsequent calcination removed PEI and TA, yielding D- or L-type chiral SiO_2_ nanofibers. To enhance the interaction with biomolecules like amino acids, the surfaces of chiral nanofibers were modified with polydopamine (PDA) through dopamine polymerization. PDA’s strong adhesive properties and versatile functional groups (amine, phenolic hydroxyl, π-π conjugation) facilitated interactions with analytes. The resulting chiral SiO_2_/PDA nanocomposites demonstrated enantioselective interactions with amino acids, producing distinct Raman intensity variations for enantiomers (e.g., cysteine, phenylalanine, leucine). Chiral recognition specificity was significantly higher (up to 2.4-fold) for D- or L-type SiO_2_/PDA composites compared to achiral silica/PDA or racemic materials (Figure 5).

This proof-of-concept study demonstrated a promising up to 2.4-fold intensity difference for enantiomers, highlighting the potential of chiral SiO_2_/PDA composites for enantioselective sensing [34]. To establish this as a reliable analytical method, future work should include statistical validation across multiple batches, investigations into the effects of ionic strength and competitive molecules, and the use of a wider range of analyte concentrations.

### 3.2. Applications in Chiral Separation

Chiral silica materials exhibit excellent thermal stability (retaining chirality after calcination at ~1000 °C) and high environmental tolerance, making them suitable for extreme separation conditions [35,36,37,38,39]. Preliminary studies suggest that chiral MSNs can exhibit remarkable thermal stability, with one report indicating the retention of chiral structural features as observed by electron microscopy after calcination at temperatures up to 1000 °C [19]. However, quantitative spectroscopic evidence (e.g., CD retention ratio) under controlled atmospheric conditions is still needed to fully corroborate the preservation of chiroptical activity at such extreme temperatures.

Their high specific surface area, tunable pore size, and ability to undergo surface functionalization with chiral groups like L-/D-tartaric acid or cysteine enable efficient enantiomer separation through the mechanisms of steric hindrance, stereoselective interactions, pore confinement [40]. Helical pores or chiral cavities selectively match enantiomers based on geometric differences. Non-covalent interactions (hydrogen bonding, π-π stacking, electrostatic attraction) between surface-modified chiral ligands and enantiomers enhance selectivity. Tortuous pores restrict diffusion rates of enantiomers, facilitating separation.

**Figure 6:**
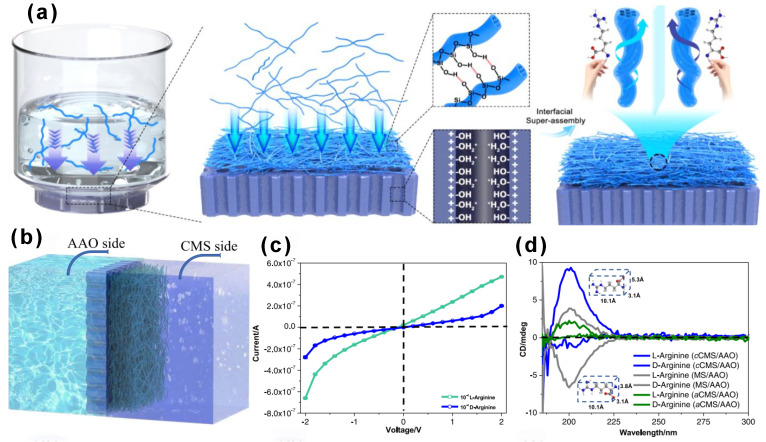
Synthesis and characterization of the cCMS/AAO heterostructured chiral membrane. Enantioselective permeation through the heterostructured chiral membrane of cCMS/AAO. (**a**,**b**) Schematic illustration for the enantioselective recognition under a certain concentration gradient, the amino acid solution with 10^−3^ M was placed on the right conductance cell, and Milli-Q water was placed on the left conductance cell. (**c**) I–V tests of the cCMS/AAO with the addition of 10^−4^ M L- and D-arginine. (**d**) Circular dichroism (CD) result for the permeated L- and D-arginine after chiral resolution of cCMS/AAO compared with aCMS/AAO and MS/AAO in 10^−4^ M L- and D-arginine, respectively. Reproduced from ref. [41] with permission from the ACS.

Kong’s group developed an asymmetric heterogeneous chiral membrane for enantiomer recognition and separation [41]. This membrane was constructed by superassembling chiral mesoporous silica (CMS) onto a porous anodic alumina (AAO) support. The CMS was prepared using folic acid (FA) as a chiral template via supramolecular chiral transcription. The asymmetric structure and chiral characteristics of the membrane enabled selective permeability and ion transport, achieving effective separation of amino acids such as L- and D-arginine. The membrane also exhibited good stability, reusability, and tunable surface properties, making it a promising platform for intelligent enantiomer separation (Figure 6). The asymmetric CMS/AAO membrane demonstrated the ability to differentiate between L- and D-arginine via I–V measurements, showcasing a promising platform for enantiomer separation [41]. To objectively evaluate its effective separation performance, future studies should benchmark it against other state-of-the-art chiral separation membranes under standardized pH and ionic strength conditions, reporting key metrics such as enantioselectivity (α) and permeability.

**Figure 7:**
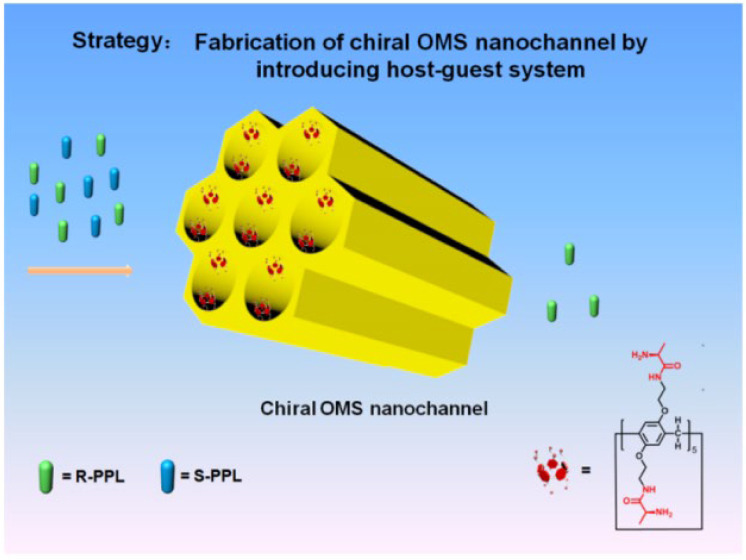
Schematic of the Design of the Chiral OMS Nanochannel-Based Host–Guest System for Enantioselective Separation. Reproduced from ref. [47] with permission from the ACS.

Chiral MSNs can serve as stationary phases for enantiomer separation in liquid chromatography (HPLC) [42,43,44] and gas chromatography [45]. For instance, grafting chiral pillar[5]arene onto silica gel microspheres created chiral recognition sites (Figure 7) [46]. This configuration achieved high separation efficiency (separation factor > 1.5) for enantiomers of drugs like alanine and valine. Remarkably, the system maintained >90% separation efficiency even after 20 reuses. Mechanistic studies revealed that the cavity structure of pillar[5]arene facilitated host-guest complexation with chiral molecules, while phenethylamine-modified silica enhanced hydrogen bonding interactions. Hong et al. prepared DNA nanoflower (DNF)-based chiral capillary silica monoliths (CSMs) by incorporating DNFs as chiral selectors [47]. These monoliths achieved successful separation of atenolol, tyrosine, histidine, and nefopam, with resolutions exceeding 1.78. Molecular docking simulations revealed that the stereoselective binding of DNA sequences enabled efficient chiral separation. The high specific surface area and binding capacity of DNFs, combined with the permeability of silica monoliths, significantly enhanced separation efficiency.

Wang et al. incorporated chiral MSNs into a polyvinylidene fluoride (PVDF) matrix to create an ultrafiltration membrane [48]. This membrane achieved 73% separation efficiency for DL-tryptophan while improving water flux (2.4×) and antifouling properties. The chiral silica pores enhanced affinity for L-tryptophan while restricting D-tryptophan diffusion, demonstrating its potential for selective separations.

Cross-comparison of these systems (Table 2) reveals several emerging trends. Chromatographic stationary phases (e.g., pillar[5]arene-silica) typically achieve high enantioselectivity (α) but may suffer from slow diffusion kinetics. In contrast, membrane-based systems offer continuous operation and scalability, though their absolute separation efficiency for a single pass can be lower. The performance is intricately linked to structural parameters: a higher density of accessible chiral recognition sites (e.g., in DNA nanoflowers) generally enhances resolution (Rs), while the pore size and connectivity of the silica support govern the mass transport and thus the efficiency. This comparison underscores that there is no single ‘best’ material; rather, the choice depends on the specific application requirements, balancing between high purity (favoring high α/Rs) and high throughput (favoring membrane processes).

### 3.3. Application in Nanomedicine

**Figure 8:**
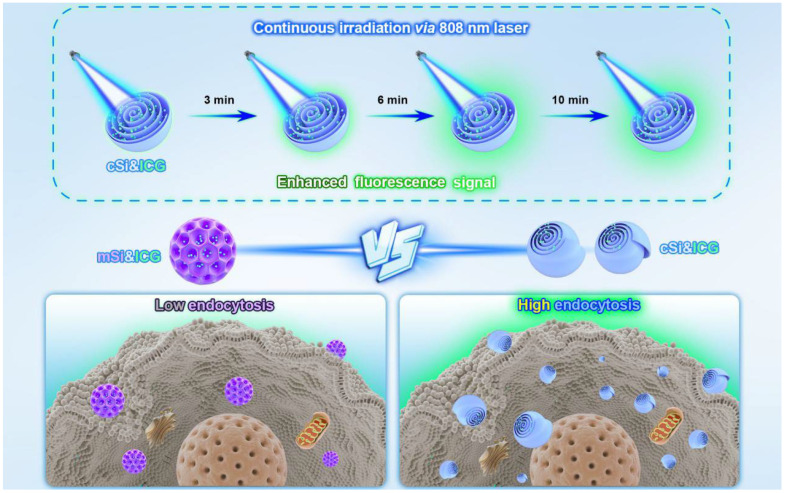
Diagram illustration of the synthesis procedure for chiral mesoporous silica with ICG loading which present higher endocytosis and enhanced fluorescence signal under continuous 808 nm light illumination. Reproduced from ref. [50] with permission from the ACS.

Chiral silica nanomaterials have shown promising applications in disease therapy, particularly in cancer therapy [49], targeted treatment, and inflammation modulation, owing to their unique chiral properties, high drug loading capacity, controlled release, biocompatibility, and low toxicity, as demonstrated in studies on Alzheimer’s disease (AD) and inflammatory bowel disease (IBD). Li et al. synthesized multi-shelled mesoporous silica nanocarriers (chiral Si nanocarriers) via a biphasic stratification reaction system (Figure 8) [50]. The unique chirality of the silica nanospheres arises from lamellar micelle spiral self-assembly at the water/oil bio-interface, differing from conventional dendritic mesoporous silica nanoparticles. Loaded with the clinical dye molecule ICG, the nanocarriers exhibit strong photostability under daylight or 808 nm laser irradiation. Their long-off peak fluorescence in the second near-infrared window (NIR II, 1000–1700 nm) is beneficial for long-duration surgical navigation. The chiral architecture enables the nanocarriers to show superior cellular uptake compared to traditional mesoporous silica. Chiral Si nanocarriers accumulate in tumor tissues and synergistically promote photothermal therapy, leading to chiral-specific killing of tumor cells and effective suppression of malignant tissues.

**Figure 9:**
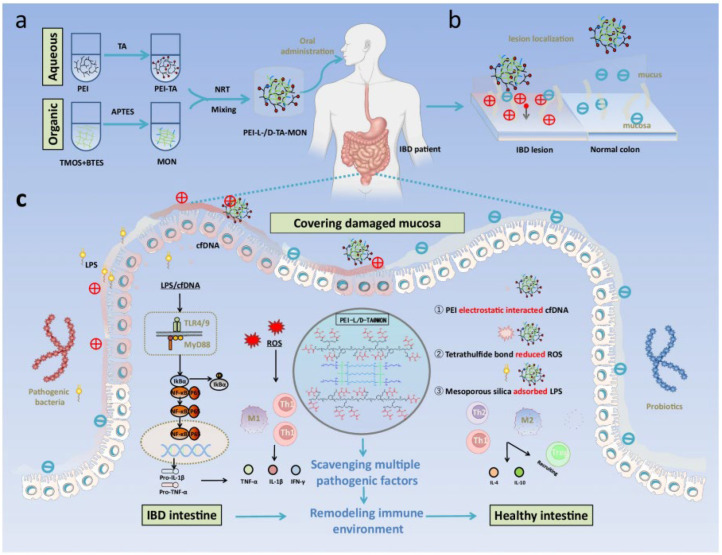
Schematic illustration on PEI-L/D-TA@MON in treating inflammatory bowel disease (IBD). (**a**) A biosilicification-mimicking route to form chiral nanostructures PEI-L/DTA@MON. (**b**) The lesion localization strategy for targeted delivery of PEI-L/DTA@MON. (**c**) Mechanisms on the generation (inflammatory cascade driven by the LPS, cfDNA and ROS) and prevention (scavenging pathogenic factors to suppress inflammation) of IBD. Reproduced from ref. [51] with permission from the Nature.

Xu et al. constructed mesoporous organosilica nanoparticles (MON) templated with PEI and L/D-TA complexes (PEI-L/D-TA@MON) as chiral nanomedicine for IBD (Figure 9) [51]. After oral administration, PEI-L-TA@MON exhibited a preferred conformation that stereochemically matched the mucosa and anchored onto the inflamed intestines, enabling targeted delivery to the lesion site. PEI-L-TA@MON could scavenge lipopolysaccharide, reactive oxygen species, and cell-free DNA, alleviate oxidative stress, suppress the inflammatory cascade, and maintain immune homeostasis, thereby achieving therapeutic effects for IBD. Furthermore, the rapid synthesis, low cost, energy-free preparation process, negligible toxicity, satisfactory therapeutic efficacy, and easy conversion of treatment modalities of PEI-L-TA@MON will bring revolutionary changes to the treatment of IBD, holding significant research value and clinical translation prospects.

**Figure 10:**
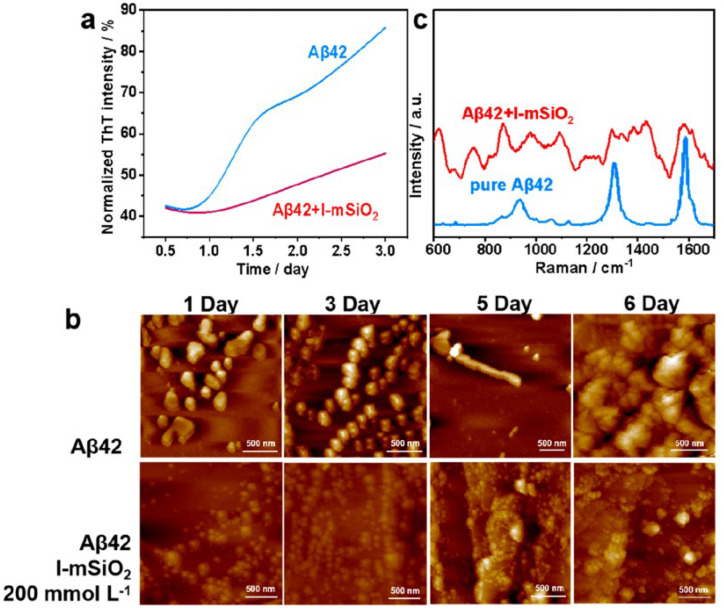
(**a**) ThT fluorescence assay, (**b**) AFM images, and (**c**) Raman spectra of Aβ42 in the absence and presence of L-mSiO_2_ nanospheres after co-incubation at different times. Reproduced from ref. [19] with permission from the ACS.

AD is a neurodegenerative disorder characterized by the abnormal accumulation of β-amyloid (Aβ) proteins, leading to neuronal damage and cognitive decline. Preventing Aβ aggregation is critical for AD prevention and treatment; however, existing small molecules, proteins, peptides, and nanomaterials exhibit limitations in inhibiting Aβ aggregation. To address this, Zhao’s group investigated the mitigation of Aβ aggregation in AD through chiral MSNs [19]. The chiral MSNs with molecular-scale chirality were successfully synthesized, featuring a radially oriented large mesoporous structure with pore sizes of approximately 10.1 nm, high pore volumes (~1.8 cm^3^/g), and high specific surface areas (~525 m^2^/g). The chiral structure of these mSiO_2_ nanospheres remained stable even after high-temperature calcination (up to 1000 °C). The chiral MSNs reduced Aβ42 aggregate formation by 79% and inhibited Aβ42-induced cytotoxicity (Figure 10). By specifically targeting the central hydrophobic segment of Aβ42, the chiral MSNs suppressed Aβ42 aggregate formation. Chiral MSNs, through their unique molecular chiral structure and open mesoporous architecture, effectively inhibit Aβ42 aggregation and alleviate Aβ-induced cytotoxicity. This discovery opens new avenues for developing novel chiral MSNs for optical and biomedical applications.

These pioneering studies reveal the tremendous therapeutic potential of chiral MSNs in treating complex diseases like AD and IBD [19,51]. It is important to note, however, that these findings are primarily from in vitro or preliminary animal models. A critical next step for clinical translation involves comprehensive in vivo pharmacokinetics, biodistribution, long-term toxicity studies, and an assessment of potential immune responses.

### 3.4. Application in Drug Delivery

The superior performance of chiral MSNs in drug delivery stems from multiple stereoselective mechanisms operating at biological interfaces. First, chiral surfaces exhibit differential interactions with phospholipid bilayers, with L-configured MSNs showing enhanced membrane permeability due to complementary stereochemistry with natural phospholipids. Second, protein corona formation is chirality-dependent, with specific serum proteins showing preferential adsorption on different enantiomorphic surfaces, subsequently influencing cellular recognition and uptake. Third, receptor-mediated endocytosis pathways often exhibit chiral preferences, with certain membrane receptors showing higher affinity for particular chiral configurations. These mechanisms collectively contribute to the observed enantioselectivity in biodistribution, therapeutic efficacy, and pharmacokinetic profiles.

In the field of drug delivery, chiral MSNs have demonstrated extensive research and application potential due to their unique structures and properties [52,53,54,55,56]. The high specific surface area, high pore volume, and tunable pore sizes of chiral MSNs make them high-performance drug uptake and sustained release [57,58,59]. Their twisted pore structures enhance drug loading capacity, and by altering pore sizes, the drug release rate can be effectively controlled. Chiral MSNs can transform drugs from crystalline to amorphous states, thereby significantly improving drug solubility and bioavailability [60,61,62,63]. Chiral MSNs exhibit good biocompatibility and biodegradability, resulting in low toxicity during in vivo applications. The chiral nature of MSNs enables them to exhibit specific chiral recognition functions in chiral environments. This characteristic allows chiral MSNs to differentiate between different enantiomers when delivering chiral drugs, thereby enhancing drug efficacy and reducing adverse effects. Chiral MSNs have used for delivering many kinds of drugs such as indomethacin [64], doxorubicin [65,66], nimesulide [67,68], flurbiprofen [69], and carvedilol [70].

**Figure 11:**
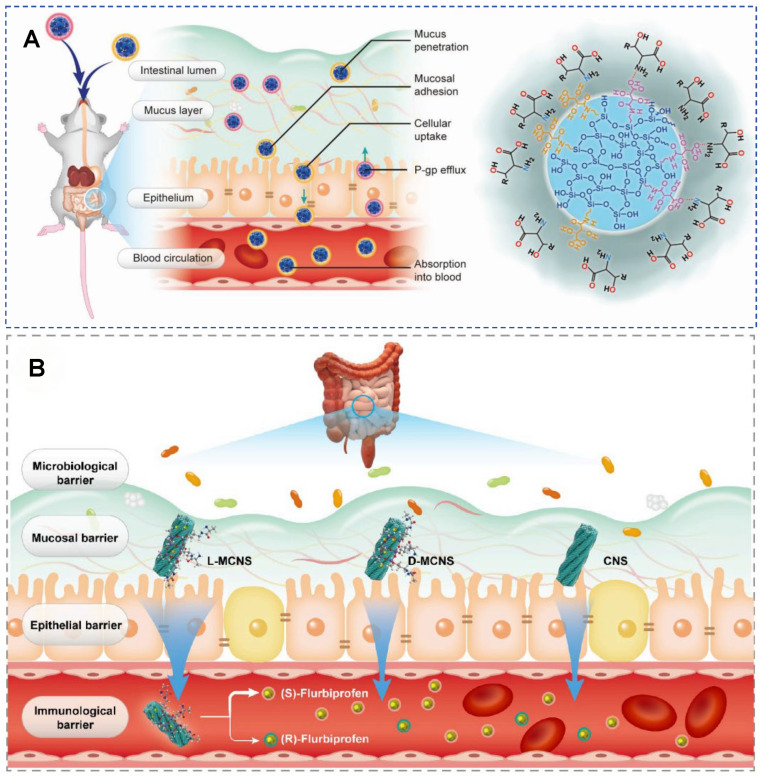
(**A**) Schematic illustration of the major barriers and main processes on the oral adsorption of L/D-tartaric acid modified mesoporous silica nanoparticles (CMSNs). Reproduced from ref. [73] with permission from the ACS. (**B**) Schematic illustration of the major barriers and main processes on the oral adsorption and blood distribution of flurbiprofen (FP) loaded chiral silica nanoscrew (CNS) and L/D-mesoporous CNS (MCNS). Reproduced from ref. [74] with permission from the ACS.

In oral delivery, the small intestinal mucosa serves as both the primary site for drug absorption and a major physiological barrier to its effective entry into the bloodstream [71,72]. In the digestive tract, nearly all structural components are chiral. In such a chiral environment, bacteria with hierarchical chirality exhibit excellent penetration, adhesion, colonization, and invasion capabilities. In 2023, Wang et al. investigated whether chiral mesoporous silica nanoparticles (CMSNs) enhance oral absorption (Figure 11A). Chiral l/d-tartaric acid-modified mesoporous silica nanoparticles (l/d-CMSNs) were prepared via a one-pot cocondensation method. The racemic (dl-CMSN), and achiral (MSN) nanoparticles were used for comparison. l-CMSN outperformed other nanocarriers in sequential oral absorption processes: mucus permeation, mucosal bio-adhesion, cellular uptake, intestinal transport, and gastrointestinal tract (GIT) retention. Electrostatic interactions dominated the chiral recognition at biointerfaces. l-CMSN showed high stability, biocompatibility, and biodegradability. When loaded with doxorubicin (DOX), l-CMSN achieved 1.72–2.05-fold higher blood absorption than other nanocarriers and improved intestinal transport of DOX by 2.32–27.03-fold. The reported 1.72–2.05-fold higher blood absorption and dramatically enhanced intestinal transport underscore the unique advantages of chiral MSNs, particularly L-forms, in oral drug delivery [71,73]. While these results are highly encouraging, the field would benefit from systematic comparisons with other leading oral nanocarriers. Furthermore, detailed investigations into potential adverse effects, such as transient intestinal stress or disruption of the mucus layer, are essential to ensure their biological safety.

In 2024, the same group developed mesoporous chiral silica nanoscrews (MCNS) for the oral delivery of flurbiprofen (FP) (Figure 11B) [74]. The chiral silica nanoscrews with a small segment area, relatively rough surface, and larger three-dimensional external surface, were modified with L- or D-type alanine isomers, achieving positive and negative chiral recognition through grafting chiral isomers. Moreover, FP@L/D-MCNS exhibited selectivity in the release of chiral drugs. Since the pharmacological activity of nonsteroidal anti-inflammatory drugs mainly originates from their S-enantiomers, the anti-inflammatory efficacy of FP@L-/D-/DL-MCNS was evaluated. Various physiological indicators and histopathological staining confirmed that FP@L-MCNS had better anti-inflammatory efficacy. Chiral MSNs exhibit enantioselectivity in oral absorption, with L-type MSNs demonstrating superior bioavailability compared to D-type and racemic forms in oral absorption. After oral administration, the penetration efficiency of L-MSNs in the intestinal mucosa is three times that of D-type, significantly enhancing the plasma concentration of levofloxacin. This may be related to the selective endocytosis of L-type MSNs by receptors on the surface of intestinal mucosal cells [71].

A systematic overview of chiral MSNs in drug delivery (Table 3) highlights their dual functionality: as high-capacity nanocarriers and as enantioselective platforms. While loading capacity is often high (frequently > 12%), it shows no clear correlation with carrier chirality, being more dependent on pore volume and drug-carrier interactions. The most significant chiral effect is observed in pharmacokinetics and targeting. L-type MSNs consistently demonstrate superior performance in oral absorption and mucosal penetration, as quantified by the enhancements in blood concentration. This suggests that the enhanced effect is not merely a marginal improvement but a statistically significant advancement attributable to the specific chiral topology’s interaction with biological barriers. Future optimization should focus on standardizing the reporting of key pharmacokinetic parameters like AUC and C_max_ to facilitate more direct comparisons.

In summary, the biological performance of chiral MSNs is profoundly influenced by their hierarchical chiral structures. At the cellular level, nanoscale chirality affects membrane interactions and subsequent internalization pathways. L-type MSNs consistently demonstrate enhanced cellular uptake across various cell lines, attributed to their preferential interactions with chiral components of cell membranes and proteins. At the tissue level, macroscopic chiral morphology influences biodistribution and targeting efficiency through stereospecific recognition by biological barriers. This multi-level chirality-function relationship underscores the importance of coordinated chiral design across molecular, nanoscale, and microscale dimensions for optimizing biomedical applications.

### 3.5. Application in Circularly Polarized Luminescence

**Figure 12:**
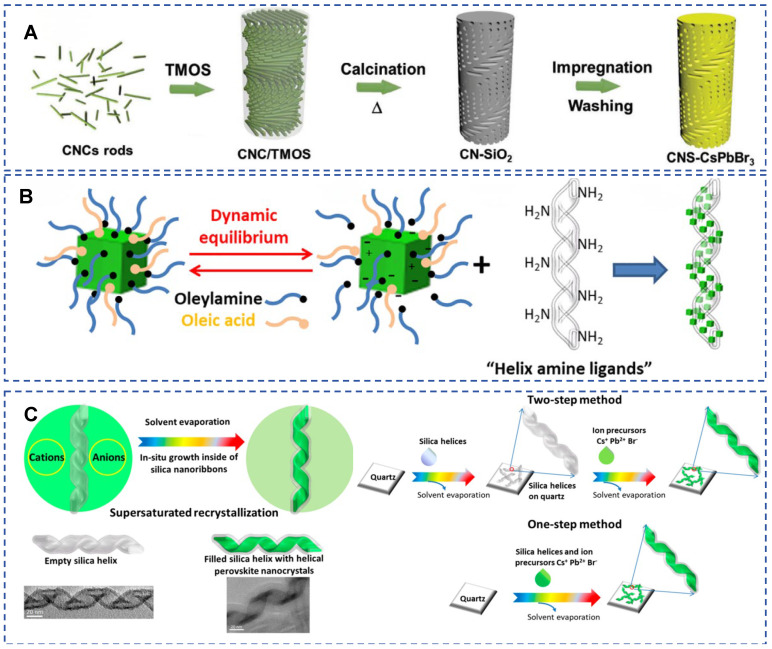
(**A**) Schematic diagram of the template-synthesis of CsPbBr_3_ confined in the mesoporous silica film with a chiral nematic structure. Reproduced from ref. [80] with permission from the Wiley-VCH GmbH. (**B**) Schematic diagram of the inorganic silica right (or left) handed nanohelices are used as chiral templates to induce optically active properties to CsPbBr_3_ PNCs grafted on their surfaces. Reproduced from ref. [75] with permission from the ACS. (**C**) Scheme of the H-PNC growth inside the hollow silica helical ribbons through one-step or two-step drop casting methods and the TEM images of empty and PNC filled silica helices. Reproduced from ref. [81] with permission from the ACS.

By encapsulating luminescent molecules or nanocrystals, chiral silicon-based nanomaterials can enhance or regulate circularly polarized luminescence (CPL) signals (Figure 12) [13,75,76,77,78]. Precise control over chiral optical properties can be achieved through solvent selection [79], drying temperature [75], or template design (such as helical hollow nanobelts, mesoporous structures) [13]. For instance, Sakai et al. encapsulated achiral luminophores in chiral silica nanocavities with helical structures to induce CPL through solvent molecule regulation, and the CPL wavelength can be adjusted by solvent selection [79]. The advantage lies in the inorganic nature of the template (solvent resistance, high-temperature stability), overcoming the drawback of organic templates being easily damaged.

Using chiral MSNs as rigid templates can induce CPL activity in fluorescent nanomaterials such as lanthanide ions [78], perovskite nanocrystals (PNCs) [80,81] or CdSe nanocrystals [76]. Liu et al. utilized chiral silica helical nanobelts as templates to grow chiral CsPbBr_3_ PNCs via supersaturated recrystallization (Figure 12C) [81]. The chiral shape of the perovskite nanocrystals originates from the spatial confinement of the silica helical nanobelt cavity, without the involvement of chiral ligands. The dissymmetry factor (g-factor) is as high as approximately ±2 × 10^−2^, significantly higher than previous perovskite systems based on chiral ligands or lattices (g ≈ 10^−3^). Confined growth within the silica template cavity significantly enhances the fluorescence intensity of perovskite nanocrystals, with stability lasting up to 3 months (high tolerance to external environments), laying the foundation for the development of chiral optoelectronic devices. For practical device applications, the environmental stability (e.g., towards heat, moisture) and reproducibility of this CPL performance across large areas are key parameters that require further evaluation and reporting.

Liu et al. grafted CsPbBr_3_ PNCs onto the surface of inorganic silica nanohlices (right-handed or left-handed) as chiral templates. In the dried film state, PNCs exhibit strong CD and CPL signals, with a dissymmetry factor (g-factor) of 6 × 10^−3^, while no chiral optical signals are observed in suspension (Figure 12B) [75]. CD and CPL originate from the tight helical arrangement of PNCs during the drying process, with the swollen ligand layer hindering electromagnetic interactions in suspension, leading to the disappearance of optical activity. Furthermore, the same group grew chiral helical perovskite nanocrystals (H-PNCs) inside helical hollow silica nanobelts via supersaturated recrystallization [81]. The CD and CPL signals of H-PNCs are determined by the shape of the silica nanobelts, and simulations confirmed that chirality originates from the geometry of the nanocrystals. Chiral mesoporous silica (CMS) can be used as templates to confine CsPbBr_3_ nanocrystals, achieving the CsPbBr_3_@CMS composite material, which exhibits high stability and strong CPL response, making it suitable for blue light-emitting diodes [77].

Zhang et al. combined natural cellulose and silica to prepare chiral nematic nanoporous silicon films, utilizing photonic band gaps to regulate phosphorescent chirality and wavelength, achieving ultra-long room-temperature phosphorescence emission (afterglow lifetime up to seconds), suitable for anti-counterfeiting labels [82].

These studies focus on optoelectronic devices (such as LEDs, 3D displays) and functional materials (such as anti-counterfeiting labels), highlighting the potential applications of chiral silicon-based materials, such as ultra-long phosphorescent films and high-efficiency blue light devices (CsPbBr_3_@CMS). This series of studies demonstrates the central role of inorganic chiral silicon-based templates in regulating the optical activity of semiconductor nanomaterials, revealing the relationship between structural design, material state, and performance, and providing theoretical and experimental foundations for the development of chiral functional materials.

### 3.6. Other Applications

Besides the above applications, chiral MSNs also hold significant promise for applications in environmental remediation, antimicrobial and asymmetric catalysis fields. For environmental applications, the increasing use of pesticides has raised environmental and public health concerns, particularly for chiral pesticides, which can exhibit toxicity even at low concentrations. Alharthi et al. synthesized chiral MSNs for the adsorption of the chiral pesticide Sulfoxaflor from water [83]. These MSNs, prepared using quaternary ammonium silane, tetramethoxysilane, proline, and cellulose as chiral selectors, achieved a removal efficiency of 98.5% under optimal conditions. The adsorption process followed a pseudo-second-order kinetic model, with an adsorption capacity of 435.45 mg/g. After multiple regeneration cycles, the removal efficiency decreased by only 10%, indicating the high reusability and cost-effectiveness of chiral MSNs for environmental remediation.

Yan’s group developed a pH-responsive chiral MSNs by loading D-Cys onto the surface of mesoporous silica nanoparticles and modifying it with polyethyleneimine molecules, achieving pH-responsive drug release [84]. D-MSNs exhibits a higher drug release rate in lower pH environments (e.g., pH 5) and a lower release rate in higher pH conditions (e.g., pH 6–7), facilitating rapid bacterial inhibition in bacterial growth settings. The strong antimicrobial capability of D-MSNs is attributed to the synergistic effect of the mesoporous structure of MSNs and D-Cys molecules. D-Cys exerts its antimicrobial effect by damaging bacterial DNA and proteins. As a pH-responsive antimicrobial material, D-MSNs effectively suppresses the growth of various bacteria and demonstrates excellent biocompatibility with mammalian cells. This study offers novel insights for developing multifunctional nanomaterials for pH-responsive antibacterial therapy.

In the catalysis, chiral silicon nanostructures can serve as substrates [85,86,87,88,89]. For example, chiral SiO_2_ nanoribbons can used for the adsorption of gold (Au) and titanium dioxide (TiO_2_) nanoparticles, forming chiral SiO_2_@Au@TiO_2_ photocatalysts with chiroptical activity [86]. The chiral silicon nanoribbons, synthesized through silane polycondensation on organic structures, were functionalized to covalently graft Au nanoparticles, creating chiral SiO_2_@Au hybrids. Subsequently, TiO_2_ nanoparticles were assembled onto SiO_2_@Au, forming chiral SiO_2_@Au@TiO_2_ hybrids with a Schottky barrier. The chiroptical activity of SiO_2_@Au@TiO_2_ hybrids influences the generation and separation of hot carriers, affecting photocatalytic efficiency. When the helicity of circularly polarized light matched the chiral SiO_2_ nanoribbons’ handedness, the photodegradation rate of rhodamine B significantly increased, highlighting the potential of chiral SiO_2_@Au@TiO_2_ hybrids in photocatalysis, particularly in reactions requiring polarization sensitivity. Through the reaction between L-proline methyl ester and N-methyl aminopropyl silica without protecting/deprotecting groups, a chiral silica-based catalyst, L-proline-(3° amine)-f-SiO_2_, were successfully prepared. This catalyst, verified by various techniques, remarkably promotes asymmetric aldol reactions at room temperature, achieving 100% conversion and >99% enantioselectivity for the S-isomer, and can be recycled seven times without activity loss [87].

## 4. Summary and Outlook

Chiral silicon materials, with their unique physicochemical properties and chiral characteristics, hold broad application potential across multiple fields. Various synthesis methods for preparing silicon materials with specific chiral structures have been developed, including template-directed self-assembly, chirality transfer strategies, and biomimetic mineralization.

While this review has summarized experimental progress, a forward-looking perspective must acknowledge the growing role of theoretical and computational methods in deciphering chirality transfer. For example, molecular dynamics (MD) simulations can model the initial stages of chiral surfactant self-assembly, revealing how molecular conformation influences micellar curvature and ultimate helical pitch. Density Functional Theory (DFT) calculations can elucidate the binding energetics and chiral induction at the ligand-nanoparticle interface. Furthermore, establishing quantitative relationships between structural descriptors (e.g., pore size, helical pitch from TEM tomography) and chiroptical properties (e.g., CD signal intensity and position) remains a crucial and emerging frontier. Such models are essential to move from empirical synthesis to the rational design of chiral MSNs with predictive control over their properties.

Chiral silicon materials show great application potential in many fields. In chiral recognition and separation, they can be used to separate enantiomers, purify chiral compounds, and are used in chromatographic separation and membrane filtration. In drug delivery systems, they exhibit good biocompatibility and bioavailability, enhance interactions with biological interfaces, promote intestinal mucosa penetration or tumor targeting, improve drug efficacy, and reduce side effects. They also excel in optical activity, producing circular dichroism and circularly polarized luminescence, making them suitable for developing optical sensors and displays. In asymmetric catalysis, they perform well as chiral catalyst supports, improving product optical purity. In the future, multifunctional chiral silicon materials integrating catalysis, chiral recognition, and drug delivery could expand their applications.

Despite significant progress, challenges remain. First, precisely controlling chiral structures and understanding chirality transfer mechanisms are ongoing challenges. The current research is in the early stages, and the chiral mechanism needs further verification, such as the molecular chirality transfer path to macroscopic helical structures. Further exploration is needed in the multiscale chiral design of CMSNs (e.g., synergistic integration of molecular/supramolecular/macroscopic chirality) and their controlled synthesis. Second, more efficient synthesis methods are needed for large-scale preparation of chiral silicon materials to reduce costs and enhance economic viability for industrial applications. Third, the biological safety and environmental impact of chiral silicon materials need to be extensively studied to ensure sustainable and safe practical applications. Fourth, improving the stability and durability of chiral silicon materials under extreme conditions is crucial for broader applications.

In conclusion, chiral silicon materials research is rapidly evolving. They have broad application prospects, especially in asymmetric catalysis, chiral recognition and separation, and biomedicine. As research deepens, chiral silicon materials are expected to play a significant role in more fields.

## Figures and Tables

**Table 1:** Comparative analysis of synthesis strategies for chiral mesoporous silica nanostructures.

Synthesis Method	Key Parameters	Resulting Chiral Structure	Advantages	Limitations	Typical Applications
Hard Template	Template morphology, Etching condition	Helical pores, Nanoscale chirality	Precise structural control	Complex process, Template removal	Chiral plasmonics, Nanomolding
Soft Template	Surfactant/Biopolymer type, Concentration, pH	Molecular-scale chiral skeleton, Mesoscale helical channels	Diversity, Tunability	Sensitivity to synthesis conditions	Drug delivery, Chiral separation
Molecular Grafting	Ligand type, Surface bonding chemistry	Molecular chiral surface (No long-range order)	Simple, Water-soluble NPs	Weak chirality, Stability issues	Chiral sensing, Bioimaging

**Table 2:** Performance comparison of chiral silica-based separation systems.

System/Material	Synthesis Method	Analyte	Key Performance Metric	Ref.
Pillar[5]arene-silica	Grafting	Alanine/Valine	Enantioselectivity (α) > 1.5	[46]
DNA nanoflower monolith	Biomolecular template	Atenolol, etc.	Resolution (Rs) > 1.78	[47]
CMS/PVDF membrane	Soft template	DL-Tryptophan	Separation efficiency: 73%	[48]
Chiral mesoporous silica	Soft template	Pharmaceuticals	Chromatographic resolution: 1.2–2.5	[43]

**Table 3:** Performance overview of chiral MSNs in drug delivery.

Drug	Drug Loading Capacity (wt%)	Release Profile	Key Enhancement	Ref.
Doxorubicin	~24%	Sustained release (~60% in 24 h, SIF); pH-responsive; chiral-dependent release	Enhanced mucus penetration, cellular uptake, intestinal transport and oral bioavailability; improved antitumor effect	[73]
Flurbiprofen	~12%	pH-dependent	Superior anti-inflammatory efficacy	[74]
Carvedilol	~19.86%	Improved sustained release	Significantly improves oral bioavailability (230.18%)	[70]
Indometacin	~32%	Chiral-recognitive release	Differential release for enantiomers	[64]

## Data Availability

No new data were created or analyzed in this study. Data sharing is not applicable to this article.
